# Low-mutation-rate, reduced-genome *Escherichia coli*: an improved host for faithful maintenance of engineered genetic constructs

**DOI:** 10.1186/1475-2859-11-11

**Published:** 2012-01-20

**Authors:** Bálint Csörgő, Tamás Fehér, Edit Tímár, Frederick R Blattner, György Pósfai

**Affiliations:** 1 Institute of Biochemistry, Biological Research Center of the Hungarian Academy of Sciences, 62 Temesvári krt, H6726 Szeged, Hungary; 2 Scarab Genomics LLC, 1202 Ann St, Madison, WI 53713, USA; 3 Department of Genetics, University of Wisconsin, 425-G Henry Mall, Madison, WI 53706, USA

**Keywords:** Escherichia coli, mutation rate, evolvability, reduced genome, synthetic biology, chassis

## Abstract

**Background:**

Molecular mechanisms generating genetic variation provide the basis for evolution and long-term survival of a population in a changing environment. In stable, laboratory conditions, the variation-generating mechanisms are dispensable, as there is limited need for the cell to adapt to adverse conditions. In fact, newly emerging, evolved features might be undesirable when working on highly refined, precise molecular and synthetic biological tasks.

**Results:**

By constructing low-mutation-rate variants, we reduced the evolutionary capacity of MDS42, a reduced-genome *E. coli *strain engineered to lack most genes irrelevant for laboratory/industrial applications. Elimination of diversity-generating, error-prone DNA polymerase enzymes involved in induced mutagenesis achieved a significant stabilization of the genome. The resulting strain, while retaining normal growth, showed a significant decrease in overall mutation rates, most notably under various stress conditions. Moreover, the error-prone polymerase-free host allowed relatively stable maintenance of a toxic methyltransferase-expressing clone. In contrast, the parental strain produced mutant clones, unable to produce functional methyltransferase, which quickly overgrew the culture to a high ratio (50% of clones in a 24-h induction period lacked functional methyltransferase activity). The surprisingly large stability-difference observed between the strains was due to the combined effects of high stress-induced mutagenesis in the parental strain, growth inhibition by expression of the toxic protein, and selection/outgrowth of mutants no longer producing an active, toxic enzyme.

**Conclusions:**

By eliminating stress-inducible error-prone DNA-polymerases, the genome of the mobile genetic element-free *E. coli *strain MDS42 was further stabilized. The resulting strain represents an improved host in various synthetic and molecular biological applications, allowing more stable production of growth-inhibiting biomolecules.

## Background

Intrinsic mechanisms for generating diversity are important for survival of bacterial populations in dynamically changing environmental conditions. The ability of a population to adapt to various situations is largely dependent upon a constant fine-tuning of mutation rate [1]. However, what is beneficial in a natural environment is not necessary when conditions are relatively stable and controlled, as in laboratory and industrial settings. In fact, novel, evolved features arising in a carefully designed and fabricated system of biological parts can lead to unwanted genotypic and phenotypic alterations, and the spontaneous genetic modification of an established production strain or a clone library is usually highly undesirable [2]. Consequently, whether used as a production strain, a cloning host, or as a synthetic biological chassis, a bacterial cell with increased genetic stability is of great importance [3-5].

In addition to being a universal cloning host, *Escherichia coli *is the most common organism used in the production of proteins, metabolites, and secondary metabolites, for both research and industrial purposes [6-12]. In an effort to improve the performance of these 'workhorse' strains, several large-scale modifications have been made to various *E. coli *strains using genome engineering methods [13-17]. These efforts all follow the basic principle of streamlining metabolic pathways for the increased production of a given biomaterial coupled with reduction of unwanted byproducts. Along these lines, a reduced-genome *E. coli *strain (MDS42) was constructed in our laboratories. Most genes irrelevant for laboratory applications, as well as all known mobile DNA sequences and cryptic virulence genes were precisely deleted, resulting in a genetically stabilized strain that displays several advantageous properties [18]. The advantages of using an MDS42 background for industrial purposes was demonstrated by increased L-threonine production in a modified version of the multi-deletion strain [19]. In a recent work, we have also shown that the IS element-free MDS42 host improves maintenance of unstable genetic constructs, allowing for stable cloning of certain toxic genes [20].

Evidence exists that some genes, in their functional forms, are unusually difficult to clone in bacterial plasmids, and aberrant clones frequently arise [21-24]. Normally, the general mutation rate of the host cell is so low (~10^-7 ^mutation/gene/generation) [25], that spontaneous changes in a cloned DNA fragment are extremely rare and therefore cannot solely account for the cloning artifacts. However, when the cloned DNA fragment interferes with normal cell physiology and reduces growth, the rare mutants of the clone can be positively selected for and can rapidly become dominant in the culture. This phenomenon became apparent to us in an earlier attempt to clone the VP60 gene of rabbit hemorrhagic virus [18]. VP60 fused to a cholera toxin component proved to be toxic to the cell. Inactivation of the recombinant gene due to IS element-transposition and insertion into the toxic gene, followed by rapid selection of the mutants, resulted in only aberrant clones in normal *E. coli *cloning hosts. Using the MDS42 host cell free of all IS elements, the recombinant gene could be cloned in its intact form.

Here we wish to expand this work by making further improvements on the genetic stability of MDS42. Beyond the previous elimination of all IS insertion events, we disabled other mutation-generating pathways of the host in order to improve tolerance and fidelity. Removal of IS elements from the host genome eliminated a major, sometimes dominant [26] form of mutation generation. However, in many cases, toxic clones are inactivated by point mutations or deletions. A detailed analysis of clones of hepatitis C virus genes showed that the cloning procedure in *E. coli *resulted exclusively in defective, non-expressing clones due to the selection of point mutants (either frameshifts or stop codons) [24]. Selection of defective forms of toxic genes can be so effective, that it can actually be used deliberately to obtain point mutants, as demonstrated by isolation of mutants of human immunodeficiency virus protease [27] or of the PvuII DNA methyltransferase [28]. Unlike insertion mutagenesis by IS elements, point mutations as a whole cannot be totally eliminated. Nevertheless, any reduction in mutation rate expands the cloning potential of the host cell and improves its function as a synthetic biological chassis.

Our strategy for reducing point mutation rate in *E. coli *involved disabling the effective mutation generating enzymes of the SOS response. Under stressful conditions (e.g. toxic clones harbored in the cell), DNA damage may occur, activating the SOS response, inducing approximately 40 members of the SOS regulon [29-32]. Three of the genes induced during the SOS response of *E. coli *encode DNA polymerases (Pol II, Pol IV, Pol V) that are able to bypass replication barriers at damaged sites and stalled replication forks [33-36]. All three of the SOS-inducible polymerases have been implicated in induced mutagenesis [37], with Pol IV and Pol V having error-rates approximately 2 to 3 orders of magnitude higher than the high-fidelity replicative polymerase (Pol III) [38]. Pol II, while showing high fidelity on undamaged templates, was shown to take part in certain types of stress-induced mutagenesis [37,39,40]. The SOS-regulated polymerases are dispensable; their primary role seems to be the generation of genetic diversity under stressful conditions [41]. DNA repair has alternative pathways, most notably recombination-mediated repair, which can rescue stalled replication in a less error-prone manner [42].

We show here that the disabling of stress-induced mutagenesis mechanisms further increases the genetic stability of MDS42, a reduced-genome *E. coli *strain lacking all mobile genetic elements. We offer proof of the beneficial effects of the resulting strain as a cloning host in the stable maintenance of a toxic gene. The improved strain shows promising potential as a cellular chassis for molecular and synthetic biological applications.

## Results

### The absence of error-prone polymerases reduces the spontaneous mutation rate

**Figure 1:**
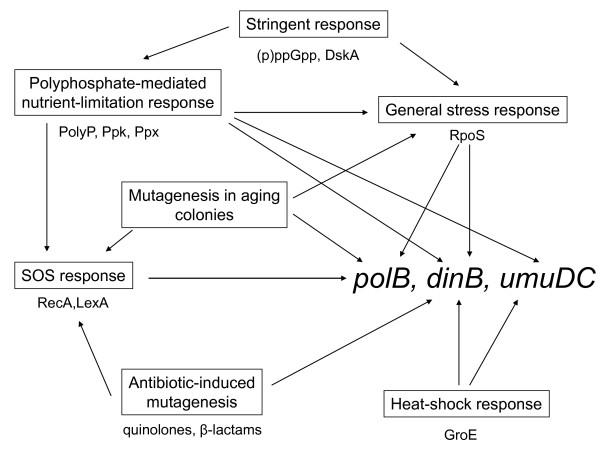
**Inducers of the error-prone polymerase genes**. Besides the well-documented SOS response-mediated pathway, error-prone DNA polymerase genes can be activated by several different routes. Main pathways are indicated in the boxes, with the main interacting partners indicated below each. (Adapted from [45]).

The SOS-induced minor (error-prone) DNA-polymerases are major factors in generating point mutations. In a published attempt, prevention of derepression of SOS regulon genes was accomplished by introducing a mutation in the primary regulator LexA, disabling the autocatalytic cleavage process involved in the regulation [43]. The mutation-decreasing effect of such a LexA variant was shown to prevent *E. coli *cells from developing antibiotic resistant topoisomerase mutations [44]. Alternatively, direct deletion of all three SOS-inducible DNA polymerase genes could reduce the mutation rate. Since these polymerases might contribute to mutation-generation even in the absence of stress, and, moreover, they can be activated by stress-induced pathways other than the SOS response [45] (Figure 1), we opted for the scarless removal of the genes encoding PolII (*polB*), PolIV (*dinB*), and PolV (*umuDC*). For comparison, LexA and RecA mutants [46], unable to induce the SOS pathway, were also analyzed.

The genes coding for the three error-prone DNA polymerases (*polB, dinB, umuDC*) were deleted from the genome of MDS42 in a scarless manner using a suicide plasmid-based method [47]. Gene deletions were made individually and also joined in all possible combinations. The spontaneous mutation rate of each strain was then determined using a D-cycloserine resistance assay, detecting all types of mutations in the *cycA *gene [48].

**Figure 2:**
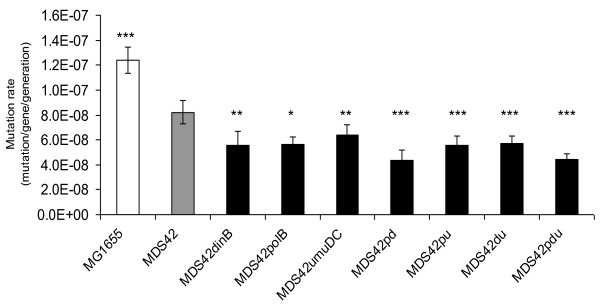
**Spontaneous mutation rates of error-prone polymerase deletion mutant and control strains**. Mutation rates were determined by the fluctuation analysis of D-cycloserine-resistant mutants [48]. Gene names are indicated for the single-deletion strains, while MDS42pd, MDS42pu, MDS42du, and MDS42pdu denote MDS42*polBdinB*, MDS42*polBumuDC*, MDS42*dinBumuDC*, and MDS42*polBdinBumuDC*, respectively. Error bars represent 95% confidence intervals for the average of 4 independent measurements. ANOVA revealed a significance of p < 0.005. Pairwise t-tests were conducted for each strain compared to the MDS42 strain, * indicates a significance of p < 0.05, ** indicates a significance of p < 0.01, *** indicates a significance of p < 0.001.

The deletion of each error-prone polymerase gene by itself results in at least a 20% decrease in mutation rate (Figure 2) measured with this method. When combining the different deletions, the mutation rate decreased further, with the lowest mutation rates being that of MDS42*polBdinB *(MDS42pd) and the triple deletion strain MDS42*polBdinBumuDC *(MDS42pdu). Compared to their parent MDS42, these strains showed a close to 50% decrease in spontaneous mutation rate (8.2*10^-8 ^mutation/cell/generation decreased to 4.34*10^-8 ^and 4.45*10^-8^, respectively). The difference observed between wild-type MG1655 and MDS42 is due to the absence of insertion events [18].

**Figure 3:**
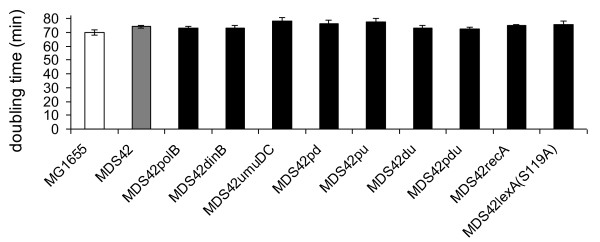
**Doubling times of strains used in the study**. Doubling times were measured in MOPS minimal medium at 37°C in microtiter plates (see methods). Error bars represent 95% confidence intervals for the average of 14 independent measurements. ANOVA revealed a significance of p < 0.005. Pairwise t-tests were conducted for each strain compared to the MDS42 strain, none of the strains showed a significant difference.

To verify that the absence of the error-prone DNA polymerases has no adverse effect on fitness, growth rates of the different strains were measured in MOPS minimal medium. 14 parallel cultures originating from 14 individual colonies for each strain were picked and grown in a Bioscreen C instrument (Figure 3). We found that none of the deletions had a significant effect on fitness in MOPS minimal medium, even when combined in the triple deletion strain MDS42pdu. This strain was then chosen for further analysis.

As an additional measure of fitness, we analyzed the survival rate of MDS42pdu and MDS42 in long-term stationary phase (Additional file 1). No significant difference was observed between the two strains over a period of 7 days. Furthermore, when the two strains were additionally stressed by expressing a moderately toxic protein from the pSin32 plasmid (discussed later), the survival rates in stationary phase were not significantly different either.

### Inactivation of the regulators of the SOS response does not lower the spontaneous mutation rate

**Figure 4:**
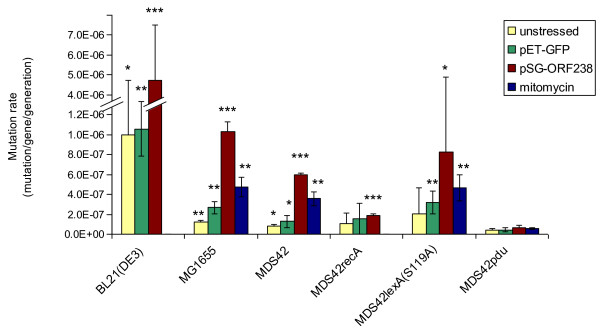
**Mutation rates of various strains under unstressed and stressful conditions**. Stress conditions include overproduction of GFP, overproduction of a toxic peptide from pSG-ORF238, and treatment with mitomycin-C. All measurements were made using the *cycA *fluctuation assay, error bars represent 95% confidence intervals for the average of 3 independent measurements. BL21(DE3) and MDS42*recA *failed to grow in the presence of 0.1 μg/ml mitomycin-C. ANOVA revealed a significance of p < 0.0001. Pairwise t-tests were conducted for each strain under a given condition compared to the corresponding MDS42pdu strain, * indicates a significance of p < 0.05, ** indicates a significance of p < 0.01, *** indicates a significance of p < 0.001.

To see whether the inactivation of the whole SOS response via regulator mutants would have the same effects on the spontaneous mutation rate as elimination of the error-prone DNA polymerases, MDS42*recA *and MDS42*lexA*(S119A) were constructed. None of these modifications had an adverse effect on the overall fitness of the strains (Figure 3). Spontaneous mutation rates of these SOS-disabled strains were analyzed by the D-cycloserine resistance fluctuation assay. Neither strain showed a significant decrease in spontaneous mutation rate when compared to MDS42 (Figure 4).

### MDS42pdu is genetically stable under stress conditions

Due to the stress-induced nature of the error-prone DNA polymerases, it was expected that the difference in mutation rates of the polymerase-free and the parent strain would be even more pronounced under stressful conditions. Mutation rates were therefore measured under stressful conditions, including the application of an antibiotic agent (mitomycin-C), overproduction of benign Green Fluorescent Protein (GFP) [49,50], and overproduction of a toxic protein (ORF238) [20].

Mitomycin-C, a DNA cross-linking agent that causes lesions in double-stranded DNA [51], directly activates the SOS response, leading to the up-regulation of error-prone DNA polymerase enzymes. A sub-inhibitory concentration (0.1 μg/ml) of mitomycin-C was used to stress the cells and analyze the effect on mutation rates. Protein overproduction imposes stress on the host cell [52,53]. To test the effect of overproduction on mutation rates, genes for either non-toxic GFP, or the toxic small, leucine-rich hydrophobic protein ORF238 [53] were cloned on plasmids as inducible constructs controlled by a T7 promoter. To express them, T7 RNA polymerase encoding variants of the studied strains were constructed. Fitness measurements of these modified strains revealed no significant decrease compared to MDS42 (Additional file 2). In addition to MDS42pdu and its parent MDS42, the widely used protein production strain BL21(DE3), the wild-type K-12 MG1655, and also the two different SOS-inactivated variants of MDS42 (MDS42*recA *and MDS42*lexA*(S119A)) were tested (Figure 4).

Results showed that, with the exception of MDS42*recA *and MDS42pdu, the various stresses generally increased the mutation rate. Overproduction of the toxic ORF238 protein had the largest effect: a > 5-fold increase in mutation rate was measured. Sub-inhibitory concentration of mitomycin-C caused a > 2-3-fold increase in the mutation rate (BL21(DE3) and MDS42*recA *were unable to grow under these conditions). The overproduction of GFP had a minor effect, a 1.5 to 2-fold increase in mutation rate.

In contrast, no significant increase in mutation rate in the presence of any of the stressors could be seen in either MDS42*recA *or MDS42pdu. (Interestingly, MDS42*lexA*(S119A) did not follow this behavior, the strain showed an increase in mutation rate in response to all of the stresses.) MDS42pdu can be characterized as the genetically most stable strain, displaying the lowest spontaneous mutation rate and showing negligible response to stressful conditions.

**Figure 5:**
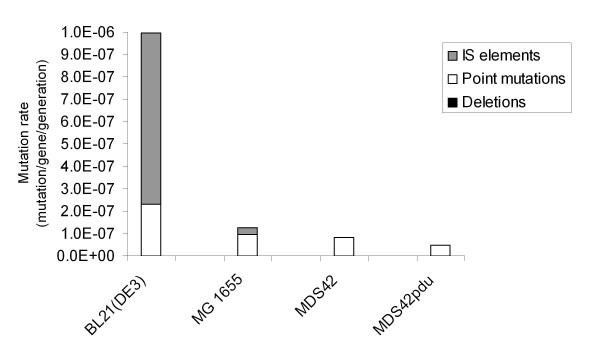
**Comparison of the mutational spectra of various strains**. The bar graph shows the distribution of *cycA *mutation types, detected by PCR analysis. The share of deletions (too low to be visible) is 1.6, 2.4, and 4.3% of the total mutations in MG1655, MDS42, and MDS42pdu, respectively. No deletions were detected in BL21(DE3).

It is also noteworthy that the commonly used protein production strain BL21(DE3) showed a mutation rate almost two orders of magnitude higher than MDS42pdu. To study what this difference could be attributed to, the mutational spectra of BL21(DE3), MG1655, MDS42, and MDS42pdu were studied by PCR analysis of *cycA *in cycloserine-resistant mutants (Figure 5). In MG1655, 74% of the mutations proved to be point mutations, 24% were IS insertions, and 2% were deletions. In contrast, in BL21(DE3), 77% of *cycA *mutations were IS insertions. Although the proportion of point mutations in BL21(DE3) was much smaller (74% in MG1655 versus 23% in BL21(DE3)), the actual rate of point mutations was also over 2-fold higher in BL21(DE3) (2.28*10^-7 ^compared to 9.2*10^-8 ^in MG1655). No deletions were found among the *cycA *alleles in BL21(DE3).

To confirm the data obtained using the *cycA *fluctuation assay, mutation rates of MDS42 and MDS42pdu under each of the different stress conditions were also measured using a second assay. The data obtained using the rifampicin resistance assay (detecting point mutations in the essential *rpoB *gene [54]) were consistent with the *cycA *fluctuation assay data (Additional file 3). MDS42pdu had a 2-fold lower spontaneous mutation frequency compared to MDS42. In response to the overproduction of the toxic ORF238 protein, as well as in the presence of mitomycin-C, the mutation rate of MDS42 became significantly elevated, while the response of MDS42pdu was much less substantial.

### The error-prone polymerase-free strain provides improved stability to a toxic protein-expressing plasmid clone

In order to demonstrate the practical advantage of working with a strain of higher genome stability, a plasmid-based mutation screen was designed. Plasmid pSin32 carries an inducible copy of s*inI*, coding for the SinI methyltransferase of *Salmonella enterica *serovar Infantis. SinI methylates the inner cytosines in DNA at GG(A/T)CC sites, producing 5-methylcytosine, thereby creating targets for the McrBC endonuclease, which cleaves DNA containing methylcytosine. A plasmid that carries methylated SinI sites (e.g., pSin32, self-methylated at its 8 SinI sites), therefore cannot establish itself in a *mcrBC+ *host. To check what ratio of an induced pSin32 sample carries mutated *sinI *(not expressing a functional SinI), the plasmid sample is transformed in both MDS42 (McrBC-) and MG1655 (McrBC+), and colony numbers are compared (see methods).

**Figure 6:**
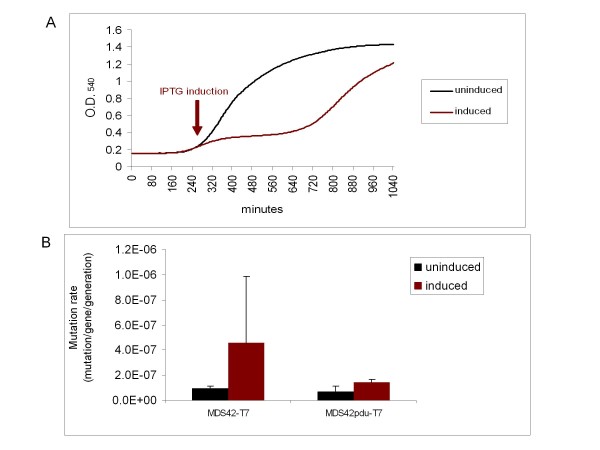
**Toxic effect of the production of SinI**. (A) Growth curves of MDS42-T7 (an McrBC- host) carrying pSin32 with and without IPTG induction. Data are averages of the O.D._540 _values of 25 independent colonies each, measured every 5 minutes using the Bioscreen C automated instrument. (B) Mutation rates of uninduced and induced (SinI-expressing) cells. Mutation rates were measured using the *cycA *fluctuation assay. Error bars represent 95% confidence intervals for the average of 3 independent measurements. ANOVA revealed a significance of p < 0.01. Pairwise t-tests were conducted for the uninduced and induced pairs resulting in a significance of p = 0.077 and p = 0.067, respectively.

BL21(DE3)*mcrBC*, MDS42-T7, and MDS42pdu-T7 were transformed with pSin32. (While MDS42-T7 and MDS42pdu-T7 are McrBC*-*, BL21(DE3) had to be specifically deleted for *mcrBC *to be able to host the plasmid.) Incidentally, it was found that, upon induction by IPTG, production of the SinI enzyme had a moderate growth-inhibiting effect even in McrBC- strains (Figure 6A). While this moderate toxicity leads to an elevation in the mutation rate of MDS42-T7, the effect is weaker (at a marginal significance) in MDS42pdu-T7 (Figure 6B), supporting the findings of the previous experiments.

**Figure 7:**
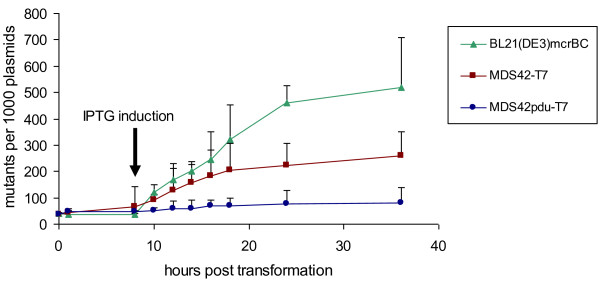
**Accumulation of plasmids with mutated *sinI *in various hosts**. SinI methyltransferase was expressed from pSin32. Plasmids were isolated at various intervals and screened (by transformation in McrBC+ and McrBC- hosts) for mutations resulting in loss of function of the enzyme. Error bars represent 95% confidence intervals for the average of 3 independent measurements of mutant plasmid ratios. ANOVA revealed a significance of p < 0.005. Pairwise t-tests of each MDS42pdu-T7 sample were done with the corresponding MDS42-T7 and BL21(DE3)*mcrBC *sample, respectively. Starting from 10 hours, all MDS42pdu-T7 samples differed significantly from the MDS42-T7 (p < 0.01) or BL21(DE3)*mcrBC *(p < 0.005) samples.

Following IPTG-induction, plasmid samples were taken at various intervals. The fraction of the plasmid sample that carried *sinI*-disabling mutations (unmethylated plasmids) was detected by transforming the plasmid samples back into MG1655 (mcrBC+). The total plasmid number per sample was determined by simultaneously transforming the samples into MDS42. After correcting each value with the transformant number from a control plasmid for each set of electrocompetent cells, the ratio of plasmids coding for functional/non-functional SinI was calculated (Figure 7).

Surprisingly, 96.7% of the starting (0-hour) plasmid sample, originating from MDS42, could not be established in MG1655. This indicated that, even in a host lacking T7 polymerase, spurious transcription of *sinI *had resulted in SinI expression, and consequently, methylation of SinI sites. The methylated status of the SinI sites in the original plasmid sample was confirmed by their uncleavability by SinI (data not shown).

Differences regarding clone stability in the different strains became evident after IPTG-induction of SinI expression. Thirty-six hours after transformation (28 hours after IPTG-induction), 51.7% of pSin32 harbored in BL21(DE3)*mcrBC *cells carried mutations preventing the production of active SinI. This value was significantly lower in MDS42-T7 (25.8%, p < 0.005 with a two-tailed, unpaired t-test). In MDS42pdu-T7, the fraction of mutated pSin32 plasmids was even lower (8.2%, p < 0.005). The non-methylated status of the SinI sites on the plasmids carrying a mutated *sinI *gene was confirmed by their cleavability by SinI (data not shown).

It seemed evident, that accumulation of mutant plasmids in BL21(DE3)*mcrBC *and MDS42-T7 was due to a combined effect of stress-induced mutagenesis and growth inhibition by the SinI-expressing plasmid. Production of the enzyme elevated mutation rates and reduced growth. In these slow-growing cultures, over time, SinI-inactivating mutations arose, which then, having resumed their normal growth rate, quickly overgrew the rest of the culture. In the low-mutation-rate MDS42pdu*-*T7, SinI-inactivating mutations developed, on average, over a longer time period. Growth curve measurements of 50 independent colonies of MDS42-T7 and MDS42pdu-T7, all carrying the pSin32 plasmid, support this notion. An O.D._540 _value of 0.7 was used as a cutoff to indicate that a culture had overcome the growth-hindering effect of the induced plasmid. The average time taken for MDS42pdu*-*T7 to reach this level of density was significantly longer than for MDS42-T7 (727.8 and 571.8 minutes, respectively; *P *< 0.005, two-tailed, unpaired *t *test).

To verify that mutations had indeed taken place in the plasmids that allowed for growth in McrBC+ cells, the *sinI *region of 8 different plasmid samples (taken from viable, pSin32-transformed MG1655 colonies) were sequenced (Additional file 4). In seven out of the eight cases, a frameshift mutation had occurred in *sinI*, resulting in a new stop codon within the gene. The eighth case displayed an A to C transversion, resulting in the N255T mutation of the protein. Six out of the seven new stop codons caused by the frameshifts were located within the first 125 bp of the gene.

## Discussion

One of the major challenges that synthetic biology must face is the intrinsic variability and genetic instability of living organisms [55]. As the complexity of synthetic systems increase, the emergence and selection of new features will become a significant impediment in achieving robust and stable performance. Improving the genetic stability of the host organism, or synthetic biological chassis is therefore a validated goal.

Previously, we have demonstrated that genome stabilization by elimination of mobile genetic elements has advantages in certain cloning applications [18,20]. To achieve additional genetic stabilization of the host, we targeted and eliminated error-prone DNA polymerases (Pol II, Pol IV, Pol V), major sources of frameshift and point mutations. Possible alternative approaches to lower the mutation rate include the introduction of a so-called antimutator *dnaE *allele or upstream inactivation of the SOS response by introducing a *recA *or *lexA *mutation. Previous studies on the effect of antimutators found a 5 to 30 fold reduction in mutation rate [56]. It was later shown that the mode of action of these DnaE antimutators was a more effective ability to exclude error-prone DNA polymerases at sites of DNA synthesis during DSB-repair associated stress-induced mutagenesis [57], suggesting that the elimination of these enzymes would reproduce the antimutator effect. Testing the other alternative approaches, MDS42*recA*, compared to MDS42pdu, showed a much less pronounced reduction in spontaneous mutation rate. Furthermore, owing to the central role of the RecA enzyme in cell physiology [58], unwanted pleiotropic effects might arise within the cell, manifested, among others, in sensitivity to mitomycin-C, presumably due to insufficient repair activity. MDS42*lexA*(S119A), carrying a non-cleavable form of the LexA repressor, did not significantly lower the mutation rate under the conditions applied. The small effect of the *recA *and *lexA *mutations can be explained by the relative SOS-independence of the error-prone polymerases: the enzymes are present even in unstressed cells, and can be up-regulated by a number of (not just SOS) stress responses (Figure 1) [45].

Several studies have been made on the effects of error-prone polymerases on mutation rates using various strains of *E. coli *and various methods of measurement. Supporting our findings is the observation that deletion of *dinB *significantly decreases the mutation rates for both frameshift and base substitution mutations in a Lac^+ ^reversion system, as well as in a rifampicin resistance assay [59]. In another study, the lack of Pol V caused a decrease in the number of Arg^+ ^growth dependent revertants [60]. Later studies also showed that post-exposure mutation rates in the presence of ciprofloxacin were markedly reduced when all three inducible polymerases were separately eliminated [44].

Here, using a D-cycloserine resistance-based fluctuation analysis [48], confirmed in some cases by a rifampicin resistance assay as well, we carefully quantified the effect of individual and combined error-prone polymerase deletions on the mutation rate, under either unstressed or stressed conditions. We determined that, under unstressed conditions, elimination of each error-prone polymerase by itself significantly decreased the spontaneous mutation rate. The effect of combining deletions Δ*polB and *Δ*dinB *was additive, indicating an independent mode of action for these polymerases. However, Δ*umuDC *generated no additional decrease of the mutation rate when any of the other two error prone polymerases was missing, possibly marking an interaction among the genes or their products. This phenomenon has been described previously regarding *ΔdinB *and *ΔumuDC*, the nature of their putative interaction, however is not yet known [61].

As expected, the most dramatic differences in mutation rate between MDS42 and MDS42pdu were observed under various stress conditions. A sub-inhibitory concentration of the SOS response-activating mitomycin-C, overproduction of either the non-toxic GFP protein or of the highly toxic ORF238 hydrophobic protein all significantly increased the mutation rate of MDS42. The values for MDS42pdu remained stable under the same conditions. It is also noteworthy, that among the strains tested, the commonly used production strain BL21(DE3) showed not just the highest spontaneous mutation rate, but also the highest increase in mutation rates in response to the various stresses. A difference of almost two orders of magnitude was observed between the mutation rate of BL21(DE3) and MDS42pdu when overproducing the toxic ORF238 protein. This elevated rate of mutation in BL21(DE3) can be mostly attributed to an increased rate of IS insertions.

A clear practical advantage of working with MDS42pdu was demonstrated in a protein production experiment, where the SinI methyltransferase was expressed from an inducible plasmid construct. SinI, producing 5-methylcytosines is toxic to cells that carry the McrBC endonuclease. Even in cells lacking McrBC, we observed a negative effect on cell fitness. When SinI was produced, we found that the *sinI *gene, carried on a plasmid, acquired loss-of-function mutations approximately three times less frequently in MDS42pdu than in MDS42, and over five times less frequently than in BL21(DE3)*mcrBC*. Remarkably, after only 16 hours of production in BL21(DE3)*mcrBC*, almost half of all *sinI *genes encoded on the plasmids had suffered a disabling mutation.

Clearly, the unexpectedly high ratio of mutated clones in the SinI-expressing culture cannot be explained solely by the stress-induced mutagenesis, the overall mutation rate of which being too low in absolute values (in the order of 10^-6 ^mutations/gene/generation) to cause such a dramatic effect. Rather, the phenomenon is in large part due to the growth inhibitory effect of the plasmid carrying the toxic gene. The chain of events is the following: Upon induction of expression of the toxic gene, growth rate of the cell is reduced. At the same time, mutation rate is increased by the stress. Once a mutant, not producing the toxic protein, arises in the plasmid population, the cell harboring it can resume normal growth and become dominant in the culture. In low-mutation-rate MDS42pdu, appearance of such mutants is delayed, and the cells can produce the functional toxic protein for an extended period of time.

Calculating the precise advantage of MDS42pdu over the parental MDS42 or the commonly used production strain BL21(DE3) can be challenging, due to the stochastic nature of mutagenesis, as well as the lack of exact data on the fitness cost of overproduction. Nevertheless, it is clear that the more severe the stress of overproducing a product is (resulting in an elevated mutation rate and growth inhibition), the greater the advantage of the stabilized host.

## Conclusions

The mutation and inactivation of engineered genetic constructs within a host cell is an overlooked problem that may have serious detrimental effects on the success of any synthetic biological, molecular biological or biotechnological process. A gene product imposing a metabolic burden or being toxic to the host drives an evolutionary force that selects for any mutants that alleviate the growth-inhibiting effect. A host cell or chassis with enhanced genetic stability is advantageous in the stable maintenance of these constructs. By eliminating the error-prone DNA polymerase enzymes from the reduced-genome MDS42 strain lacking all genomic IS elements, we have further stabilized a strain that already showed clear advantages in cloning applications. The resulting MDS42pdu strain had a significant stabilizing effect on a toxic protein expression clone. This high-fidelity strain, producing decreased genetic variation in the culture, might also prove useful in applications ranging from the production of DNA therapeutics to long-term continuous fermentation processes.

## Methods

### Strains, plasmids, media, and oligonucleotide primers

Most of the strains used in this study were constructed from *E. coli *MDS42 [18], a reduced genome strain derived from K-12 MG1655 [62]. Individual, scarless deletions (*polB*, *dinB*, *umuDC, mcrBC*) or allele replacements (*lexA*) were constructed by a suicide plasmid-based method. Standard steps and plasmids (pST76-A, pSTKST) of the procedure have been described [47]. Deletion of *recA *was carried out with a similar strategy, using plasmid pSG2857 (Scarab Genomics, Madison, Wisconsin, USA). Individual deletions were combined by P1 phage transduction [63] of the marked (with integrated suicide-plasmids) intermediates of the deletion constructs, followed by endonuclease cleavage-stimulated out-recombination and loss of the plasmid. Co-ordinates of the individual deletions are shown in Table 1. All deletions and modifications were verified by PCR and sequencing using flanking primers. T7 polymerase-expressing strain variants (MDS42-T7 and MDS42pdu-T7) were constructed by replacing the *yahA-yaiL *genomic region with an IPTG-inducible *lac *operator/T7 polymerase cassette. Plasmid pSG-ORF238 is an IPTG-inducible, pSG1144-based construct capable of overproducing the hydrophobic ORF238 toxic protein [20]. Plasmid pET-GFP is an IPTG-inducible pET-based construct carrying the *gfp *gene. Plasmid pSin32 is a pET3-His based construct [64] carrying an inducible, N-terminal His-tagged s*inI *methyltransferase gene cloned into the XhoI site of the original vector [65]. Plasmids were prepared using IS-free MDS42 host. LB and LB-agar plates were used for routine cultivation [66]. The following final antibiotic concentrations were used: 50 μg/ml ampicillin (Ap), 25 μg/ml chloramphenicol (Cm), 25 μg/ml kanamycin (Kan), 100 μg/ml rifampicin (Rp), and 4 μg/ml D-cycloserine (Cyc). For the *cycA *fluctuation assays, minimal salt (MS) medium, supplemented with 0.2% glucose, was used [26]. Growth measurements were made in MOPS minimal defined medium (Scarab Genomics, Madison, Wisconsin, USA). Additional file 5 lists the oligonucleotides used in the experiments.

**Table 1:** Genomic coordinates of each scarless deletion.

Strain	Coordinates of deletion
MDS42*recA*	2820783-2821861

MDS42*polB*	63505-65764

MDS42*dinB*	250904-251966

MDS42*umuDC*	1230261-1231298

BL21(DE3)*mcrBC*	4408095-4515388

The data regarding the MDS42-based strains are given using MG1655 coordinates.

### Growth measurements

Growth properties were evaluated in liquid medium in 100-well Honeycomb 2 plates (Oy Growth Curves Ab, Helsinki, Finland). Growth curves were measured by following the optical densities (O.D.) at 540 nm in each well using the Bioscreen C Automated Microbiology Growth Analysis System (Oy Growth Curves Ab, Helsinki, Finland). Fourteen individual colonies from each strain type were resuspended and grown in parallel to saturation at 37°C in MOPS medium. From the saturated cultures, 2 μl was transferred to 198 μl fresh MOPS medium and grown to saturation in individual wells at 37°C using continuous shaking. The median O.D. value of the fourteen parallel cultures corresponding to each strain was calculated and plotted for each time point. Doubling times were calculated from these growth curves using previously described methods [67]. For analysis of growth in the presence of the SinI methyltransferase, 50 resuspended individual colonies of MDS42-T7 and MDS42pdu-T7, each harboring the pSin32 plasmid, were grown to saturation in LB medium supplemented with 50 μg/ml ampicillin. From the saturated cultures, 2 μl was transferred to 198 μl of fresh LB and ampicillin and grown to O.D. = 0.2, then IPTG inducer was added at a final concentration of 1 mM. An O.D._540 _value of 0.7 was arbitrarily chosen as a point where the culture had overcome the toxic effect of SinI. The duration of the growth inhibition for each sample was averaged for both strains.

To measure long-term survival of individual strains, 5 ml LB cultures were inoculated 1:1000 (vol:vol) from fresh overnight cultures. Viable counts were determined directly from the cultures incubated at 37°C for up to one week.

### Mutation rate measurements

D-cycloserine resistance assays were performed as previously described [48]. Briefly, in a fluctuation assay, 20 tubes of 1 ml MS medium [26] supplemented with 0.2% glucose were inoculated with approximately 10^4 ^cells each, and cultures were grown to early stationary phase. Aliquots of 50 μl from each tube were then spread on MS plates containing D-cycloserine (0.04 mM). The number of mutations per tube (*m*) was estimated from the number of colonies by fluctuation analysis using the Ma-Sandri-Sarkar maximum-likelihood method [68]. Equation 41 from the report of Stewart *et al*. [69] was used to extrapolate the obtained *m *value, valid for 50 μl, to 1 ml. Statistical comparisons of *m *values were made only when the difference in total cell number was negligible (< 3%, *P *≤ 0.6, with a two-tailed, unpaired *t *test). The total number of cells in a tube was calculated by spreading dilutions from three random tubes onto nonselective plates. Dividing the number of mutations per tube by the average total number of cells in a tube gave the mutational rate (mutation/gene/generation). To assess the effect of the antibiotic mitomycin-C on mutation rate, 0.1 μg/ml of mitomycin-C was added to each tube. When measuring mutation rates of cells harboring a protein-overproducing plasmid, cultures were induced with 1 mM IPTG at an O.D._540 _value of 0.2. In these cases the selective antibiotic for the specific plasmid was also present in the MS medium.

In a second protocol, to confirm data obtained using the *cycA *assay, cells resistant to rifampicin (carrying mutations in *rpoB *[54]) were selected and counted. Twenty tubes of 1 ml LB were inoculated with 10^4 ^cells each, and cultures were grown to early stationary phase. Appropriate dilutions were spread onto non-selective LB agar plates and LB agar plates containing rifampicin (100 μg/ml). Colony counts were performed after 24 or 48 h, respectively. Mutation frequencies were reported as a proportion of the number of rifampicin-resistant colonies relative to the total viable count. The results correspond to the mean value obtained in three independent experiments for each strain and condition. When required, different stress conditions were provided in the same manner as in the *cycA *assay.

### Analysis of mutational spectra

Analysis of the mutational spectrum of the *cycA *gene has been described previously [48]. In brief, a 1877-bp genomic segment encompassing the entire gene was amplified from mutant cells using the primer pair cycA-D/cycA-E. A representative sample was obtained by analyzing 5 colonies from each parallel plate, yielding a total of 96 samples per experiment. The amplified fragments were resolved on an agarose gel and compared to a fragment generated from the wild-type template. Identical sizes indicated a mutation affecting only one or a few nucleotides, a decrease in size or failure of amplification indicated a deletion, and a detectable increase in size indicated an IS insertion.

### Assay to detect mutations in *sinI*

Plasmid pSin32 carries the gene *sinI *coding for SinI methyltransferase of *Salmonella enterica *serovar Infantis cloned into the XhoI site of the pET3-His plasmid [64]. The plasmid was electroporated into MDS42-T7, MDS42pdu-T7 and BL21(DE3)*mcrBC*. After 1 hour of recovery incubation at 37°C in 1 ml LB, 100 μl of the transformed cultures were placed in 100 ml LB supplemented with Ap and incubated at 37°C. From the remaining 900 μl, plasmid DNA was isolated according to standard protocols [66]. After 7 hours of incubation, the cultures reached O.D._540 _= ~0.2, at which point the samples were induced with IPTG (1 mM final concentration). Samples for plasmid preparation were also taken at this time (8-hour samples), followed by additional samples being taken every 2 hours, up to 18 hours, then at 24 and 36 hours of post-transformation growth. Purified pSin32 plasmid samples (9 from each strain) were then transformed into MDS42 (McrBC^-^) and MG1655 (McrBC^+^). By counting transformed MG1655 and MDS42 colonies for each plasmid sample, the relative number of mutated plasmids could be calculated. To obtain an absolute value for mutated plasmid numbers, each batch of electrocompetent MDS42 and MG1655 indicator strains was transformed with a control (pST76-A) plasmid [70] carrying an Ap resistance cassette. The ratio of MG1655 and MDS42 transformants was then used as a correcting factor to calculate absolute values for the number of mutated pSin32 plasmids for each sample.

## Competing interests

Frederick R. Blattner has a financial interest in Scarab Genomics LLC.

## Authors' contributions

BC performed strain development, protein overexpression and cloning experiments, mutation rate measurements, participated in experimental design, and drafted the manuscript. TF participated in experimental design, acquisition of funding, and critical reading of the manuscript. ET assisted with strain development. FRB provided intellectual help, as well as critical reading and correction of the manuscript. GP conceived the research, obtained funding, coordinated the experiments and corrected the manuscript. All authors have read and approved the final manuscript.

## Supplementary Material

Additional file 1 **shows the long-term survival rates of MDS42 and MDS42pdu in stationary phase**. Click here for file

Additional file 2 **shows the doubling times of T7 RNA polymerase containing strains used in the study**. Click here for file

Additional file 3 **shows the mutation rates of MDS42 and MDS42pdu under different conditions measured using a rifampicin resistance assay**. Click here for file

Additional file 4 **lists the mutations that occurred in eight sequenced pSin32 plasmids isolated from McrBC+ hosts**. Click here for file

Additional file 5 **lists the sequences and a short description of the PCR primers used in the study**. Click here for file
