# Supplementary material for: Low-mutation-rate, reduced-genome Escherichia coli: an improved host for faithful maintenance of engineered genetic constructs
Source: Microb Cell Fact. 2012 Jan 20;11:11. doi: 10.1186/1475-2859-11-11 (PMC3280934; doi:10.1186/1475-2859-11-11)
Supplement: Additional file 1 — shows the long-term survival rates of MDS42 and MDS42pdu in stationary phase. [file 1475-2859-11-11-S1.DOC]

**Additional file 1. Long-term survival pattern of strains in stationary phase.** Cells were grown alone in batch cultures, from which viable counts were directly determined each day. The strains carrying pSin32 were induced with 1 mM IPTG at an O.D._540_ value of 0.2 following the initial inoculation. CFU/ml: colony-forming units per milliliter.
