# Supplementary material for: Low-mutation-rate, reduced-genome Escherichia coli: an improved host for faithful maintenance of engineered genetic constructs
Source: Microb Cell Fact. 2012 Jan 20;11:11. doi: 10.1186/1475-2859-11-11 (PMC3280934; doi:10.1186/1475-2859-11-11)
Supplement: Additional file 2 — shows the doubling times of T7 RNA polymerase containing strains used in the study. [file 1475-2859-11-11-S2.DOC]

**

*

**Additional file 2. Doubling times of protein expression strains used in the study.** Doubling times were measured in MOPS minimal medium at 37˚C in microtiter plates (see methods). Error bars represent 95 % confidence intervals for the average of 14 independent measurements. ANOVA revealed a significance of p<0.001. Pairwise t-tests were conducted for each strain compared to the MDS42 strain, * indicates a significance of p<0.05, ** indicates a significance of p<0.01.
