# Supplementary material for: Low-mutation-rate, reduced-genome Escherichia coli: an improved host for faithful maintenance of engineered genetic constructs
Source: Microb Cell Fact. 2012 Jan 20;11:11. doi: 10.1186/1475-2859-11-11 (PMC3280934; doi:10.1186/1475-2859-11-11)
Supplement: Additional file 3 — shows the mutation rates of MDS42 and MDS42pdu under different conditions measured using a rifampicin resistance assay. [file 1475-2859-11-11-S3.DOC]

**Additional file 3. Mutation rates of MDS42 and MDS42pdu under various conditions, measured using a rifampicin resistance assay.** Error bars represent 95 % confidence intervals for the average of 3 independent measurements. ANOVA revealed a significance of p<0.0001. Pairwise t-tests performed between MDS42 and respective MDS42pdu mutation frequencies indicated significant differences under every test condition (* indicates a significance of p<0.05 compared to the corresponding MDS42pdu strain).
