# Supplementary material for: Low-mutation-rate, reduced-genome Escherichia coli: an improved host for faithful maintenance of engineered genetic constructs
Source: Microb Cell Fact. 2012 Jan 20;11:11. doi: 10.1186/1475-2859-11-11 (PMC3280934; doi:10.1186/1475-2859-11-11)
Supplement: Additional file 4 — lists the mutations that occurred in eight sequenced pSin32 plasmids isolated from McrBC+ hosts. [file 1475-2859-11-11-S4.DOC]

| **Sequenced plasmid** | **DNA sequence mutation** | **Resulting change in protein** |
| --- | --- | --- |
| 1 | frameshift (-1) at position 81 | stop codon at position 94 |
| 2 | frameshift (+1) at position 68 | stop codon at position 74 |
| 3 | frameshift (-1) at position 82 | stop codon at position 94 |
| 4 | A-C transversion at position 766 | N255T missense mutation |
| 5 | frameshift (+1) at position 69 | stop codon at position 74 |
| 6 | frameshift (+1) at position 748 | stop codon at position 755 |
| 7 | frameshift (-1) at position 80 | stop codon at position 94 |
| 8 | frameshift (+1) at position 94 | stop codon at position 125 |

**Additional file 4. Loss of function mutations in *sinI,* revealed by screening a pool of pSin32.** Eight pSin32 plasmid samples, able to transform MG1655 (McrBC+ host), were sequenced. Seven of these carried frameshift mutations that created new stop codons in *sinI*. One of the mutations was a A→C transversion leading to an Asn→Thr change. Nucleotide position of each mutation is shown relative to the first nucleotide of *sinI* coding region (1413 bp in length).
