# Supplementary material for: Low-mutation-rate, reduced-genome Escherichia coli: an improved host for faithful maintenance of engineered genetic constructs
Source: Microb Cell Fact. 2012 Jan 20;11:11. doi: 10.1186/1475-2859-11-11 (PMC3280934; doi:10.1186/1475-2859-11-11)
Supplement: Additional file 5 — lists the sequences and a short description of the PCR primers used in the study. [file 1475-2859-11-11-S5.DOC]

| Primer name | Sequence (5'-3') | Application |
| --- | --- | --- |
|  |  |  |
| polB-A | ccgaattcagtatccaggcgagt | deletion of *polB* |
| polB-BR | caggcaggtgtggcggagggaatact | deletion of *polB* |
| polB-BF | tccgccacacctgcctgcgccacgct | deletion of *polB* |
| polB-C | ccggatccattggcggcattgt | deletion of *polB* |
| polB-D | tgctgaacaccagtttgct | deletion of *polB* |
| polB-E | aaccggtgaagtggttga | deletion of *polB* |
| dinB-A | ccggtaccgggcataccgatgcga | deletion of *dinB* |
| dinB-BR | cagaatatacattgctcacctctcaacact | deletion of *dinB* |
| dinB-BF | gaggtgagcaatgtatattctggtgtgca | deletion of *dinB* |
| dinB-C | ccggatccgccgttaacgcatcaa | deletion of *dinB* |
| dinB-D | gtgttcgactcgctcgat | deletion of *dinB* |
| dinB-E | gagtcgtcgtagagtgcat | deletion of *dinB* |
| umuDC-A | ggaattcggatgagcgtcgtcgcca | deletion of *umuDC* |
| umuDC-BR | ttgagcgcaacaacagcagcgatgacaa | deletion of *umuDC* |
| umuDC-BF | gctgctgttgttgcgctcaatgaacctt | deletion of *umuDC* |
| umuDC-C | gctgcagatcgcttacctgattgtc | deletion of *umuDC* |
| umuDC-D | aatgctccatctgcggtt | deletion of *umuDC* |
| umuDC-E | gctctatccttcgccgtt | deletion of *umuDC* |
| lexA-A | gttatggtcgcattttggata | modification of *lexA* |
| lexA-BR | gatatctttcatcgCcatcccgctgacgcgca | modification of *lexA* |
| lexA-BF | ggatgGcgatgaaagatatcggca | modification of *lexA* |
| lexA-C | ccggatcccagcaacggaacggt | modification of *lexA* |
| lexA-D | cggtgctgattgccatta | modification of *lexA* |
| lexA-E | gggctatcaagatgacca | modification of *lexA* |
| recA-D | cggctagcgacgggatgttgattc | deletion of *recA* |
| recA-E | gtgctgattatgccgtgt | deletion of *recA* |
| BMD30-A | ccgaattcagtccgcacgcaactt | deletion of *mcrBC* |
| BMD30-BR | ctcgccttaatttacatacttttggtgc | deletion of *mcrBC* |
| BMD-30BF | tatgtaaattaaggcgagattattaaa | deletion of *mcrBC* |
| BMD30-C | ccggatccacatggcgcgttacaa | deletion of *mcrBC* |
| BMD30-D | tgataccgccgcacaaca | deletion of *mcrBC* |
| BMD30-E | actggtgtgtctcgcaag | deletion of *mcrBC* |
| cycA-D | ctgatgccggtaggttct | analysis of *cycA* mutations |
| cycA-E | gcgccatccagcatgata | analysis of *cycA* mutations |
| AK54-D | atgataatgaatgacatca | sequencing of *sinI* |
| AK55-E | ctcgagttagaccaactctccaaa | sequencing of *sinI* |
| Sce2 | attaccctgttatcccta | pST76-specific primer |
| T7 | taatacgactcactataggg | pST76-specific primer |

**Additional file 5. Sequences of the primers used in the study.**

Primers marked with A, C, BF, and BR were used to create homology regions by recombinant PCR for genomic integration of the suicide plasmids [44]. Primers marked with D or E were homologous to flanking genomic regions, and were used for checking the deletions/allele replacements by PCR and sequencing. Capital letters in *lexA* primers indicate the point mutation introduced in the gene.
